# 黑色素瘤相关抗原（*MAGE*）基因在肺癌中的表达及意义

**DOI:** 10.3779/j.issn.1009-3419.2013.06.07

**Published:** 2013-06-20

**Authors:** 广旭 李

**Affiliations:** 1 250022 济南，济南大学山东省医学科学院医学与生命科学学院 Shandong Academy of Medical Sciences, School of Medicine and Life Sciences, University of Jinan, Jinan 250022, China; 2 250117 济南，山东省肿瘤医院胸外科 Thoracic Surgery, Shandong Tumor Hospital, Jinan 250117, China

**Keywords:** 肺肿瘤, 微转移, 黑色素瘤抗原基因, 主动免疫治疗, Lung neoplasms, Micrometastasis, Melanoma associated antigens genes, Active immunotherapy

## Abstract

肺癌是常见的恶性肿瘤之一，因目前诊断易忽略微转移灶，造成肺癌预后极差，黑色素瘤相关抗原（melanoma associated antigens, *MAGE*）基因作为一种特异性肿瘤抗原基因，在肺癌的发生、发展和治疗中起着重要作用，其研究为肺癌的诊断和治疗提供了新的方向。

## Abstract (en)

Lung cancer is one of the common malignancies with an extremely poor prognosis, because of the current diagnostic techniques are not easy to find micrometastases. Melanoma associated antigens genes (*MAGE*) are tumor specific antigen genes, closely related to the occurrence, development and prognosis of lung cancer. The research of *MAGE* genes provide a new direction for the diagnosis and treatment of lung cancer.

肺癌是最常见的恶性肿瘤之一，其发病率高，预后差，与肺癌易发生血行转移和淋巴结转移有关^[1]^。淋巴转移和血行转移是肺癌的主要转移方式，淋巴结及血行转移状况对于肺癌的准确分期、治疗及预后具有重要的意义^[2]^。但因微转移灶不易被常规的检查和诊断方法发现，使肺癌分期不准确，甚至耽误了患者的治疗。因此，探寻更有优势的微转移检测指标和方法极为重要。作为一种高度肿瘤特异性相关基因，黑色素瘤相关抗原基因被认为是目前最佳的微转移检测指标，本文将就其在肺癌微转移中的研究进展做一综述。

## 1 黑色素瘤相关抗原基因（melanoma associated antigens genes, *MAGE*）基因的发现及结构

*MAGE*基因是一种原癌基因，属于肿瘤-睾丸抗原（cancer-testis antigen, CTA）家族，首先从黑色素瘤中发现，目前发现的*MAGE*基因家族成员超过60个，*MAGE*基因家族的最大共同特征是其氨基酸序列存在一个MAGE同源结构域（MAGE homology domain, MHD）。MHD一般含165个-171个氨基酸残基，根据其氨基酸排列进行的结构预测表明，它可能含有4个α螺旋和5个β片层结构，但是其真实结构及功能目前还不是很清楚。根据*MAGE*基因在染色体上具体位置的不同及其表达模式的不同，*MAGE*基因被分为两大亚家族，分别为MAGE-Ⅰ类抗原和MAGE-Ⅱ类抗原，MAGE-Ⅰ类抗原属于癌睾丸抗原中的一个大家族，其又可分为MAGE-A、B和C三个亚家族，它们主要分布在生殖细胞和滋养细胞；MAGE-Ⅱ类抗原包括MAGE-D、神经细胞生长抑制因子抗原、网状内皮系统刺激素抗原等，主要表达于神经组织和其它正常组织。在MAGE-Ⅰ类抗原中MAGE-A具有严格的肿瘤特异性表达模式，可编码肿瘤特异性抗原多肽，其编码的蛋白产物能被细胞毒性T淋巴细胞（cytotoxic T lymphocyte, CD8^+^）识别并诱导免疫应答，常被作为肿瘤诊断和免疫治疗的重要靶分子，因此成为肿瘤免疫治疗研究的热点。

## 2 *MAGE*基因的调控及表达

### 2.1 *MAGE*基因的调控机制

*MAGE*基因在除了睾丸和胎盘组织之外的正常组织中几乎不表达，但在肿瘤组织中却存在高表达现象，目前在肺癌、肝癌、肾癌、黑色素瘤等肿瘤中均检测到*MAGE*基因的表达。因其肿瘤特异性，对其研究逐渐受到重视。

目前认为*MAGE*基因表达调控机制主要有两种：①*MAGE*基因是一种正常基因，甲基化控制其表达，基因调控方式中便包括DNA甲基化这一项。通常*MAGE*基因在人类正常体细胞中高度甲基化，而在某些恶性肿瘤细胞和胚胎细胞中表现为去甲基化。因为被DNA甲基化所调控的CpG岛（CpG island）或富含CpG的区域控制着大部分的肿瘤睾丸抗原基因启动子机*MAGE-A*基因，所以抑制转录主要是通过防止转录因子的结合和增加甲基化-CpG结合区域蛋白（methylation-CpG binding region of proteins, MBDs）来实现^[3]^。②*MAGE-A*基因的表达调控与组蛋白的乙酰基化作用有关。组蛋白乙酰化可以从以下两方面促进基因转录：转录活化因子能识别氨基末端乙酰化的核心组蛋白，松弛染色质，暴露核小体DNA，利于转录因子与之结合；组蛋白乙酰化可干扰组蛋白氨基末端与抑制因子之间的相互作用。Wischnewski等^[4]^通过逆转录-聚合酶链反应（reverse transcription-polymerase chain reaction, RT-PCR）、Western杂交、免疫细胞化学染色和瞬时转染分析等方法，证实了启动子的去甲基化和组蛋白乙酰化这两种方法均可调控人肿瘤细胞*MAGE-A*基因的表达。Yanagawa等^[5]^应用RT-PCR技术对比分析了非小细胞肺癌患者的癌组织及非癌组织中MAGE-A1和MAGE-A3启动子去甲基化和其表达的关系，结果在90%表达MAGE-A1的癌组织样本中检测到MAGE-A1启动子的去甲基化，在92.3%表达MAGE-A3的癌组织样本中检测到MAGE-A3启动子的去甲基化，统计分析显示*MAGE*基因启动子去甲基化与*MAGE*基因表达之间差异有统计学意义，*MAGE*基因启动子去甲基化是诱发*MAGE*基因的表达首要原因。

### 2.2 *MAGE*基因在肺癌的表达

基于*MAGE*基因肿瘤表达特异性及抗原特异性，近年来*MAGE*基因与肺癌的相关研究逐步受到重视。Shin等^[6]^提取133例患者肺组织及痰液中的RNA，应用RT-PCR技术行*MAGE*基因检测，发现*MAGE A1-A6*在肺癌组织中的阳性率为87.5%，在肺癌患者的痰液中的阳性率为50.8%；40例*MAGE*基因阳性的病例中，33例被诊断患有肺癌，7例为肺良性疾病，但这7例中6例出现了*MAGE*基因高度甲基化保护，因此可以说明MAGE的阳性表达提示着肺癌细胞或癌前细胞的存在。Karimi等^[7]^采用RT-PCR方法检测*MAGE-A1*、*MAGE-A2*、*MAGE-A3/6*、*MAGE-A4*、*MAGE-A12*基因在肺癌中的表达，结果在58个肿瘤样本中，至少有1种*MAGE*基因表达的样本数为37个，其中MAGE-A4的表达率最高。Kim等^[8]^应用RT-PCR方法检测*MAGE A1-6*基因在53例疑似肺癌患者组织样本中的表达，结果*MAGE*基因在肺癌中的敏感性、特异性、准确性、阳性和阴性预测值分别为83%、58%、77%、87%和55%。这证明*MAGE*基因与肺癌具有高度密切性。Yanagawa等^[5]^应用RT-PCR技术对67例非小细胞肺癌患者的癌组织及非癌组织进行MAGE-A1和MAGE-A3的表达检测，结果在全部非癌组织中均未查到MAGE-A1和MAGE-A3的表达，在肺癌组织中MAGE-A1的表达率为29.9%，MAGE-A3的表达率为38.8%，MAGE-A1和MAGE-A3在鳞癌中的表达高于腺癌，随着病情进展，其表达率成逐渐升高趋势。MAGE-A1和MAGE-A3表达组患者的预后差于不表达组患者。国内相关研究也已开展，韩秀晶等^[9]^应用间接酶免疫组化技术检测MAGE-A3在45例非小细胞肺癌组织中的表达情况，结果显示MAGE-A3在非小细胞肺癌中的敏感度为62.2%，与Yanagawa的观点不同，其检测出MAGE-A3在腺癌中的表达率高于鳞癌，与性别、年龄、淋巴结转移无关。*MAGE*基因在肺癌组织中的表达这方面，国内外学者已达到共识，但关于癌型及临床分期与*MAGE*基因表达的联系存在不同的观点，Shin等^[10]^应用PCR法检测75例肺癌患者组织中*MAGE A1-A6*基因的表达，发现*MAGE*基因的表达与肺癌组织类型和临床分期无相关性，仅与肿瘤大小有关，在 < 3 cm的肿瘤中其检出率为74%， > 3 cm的肿瘤中检出率为58.7%。综上所述，*MAGE*基因在肺癌中的表达有以下规律：①*MAGE*基因在肺癌中的表达具有高度特异性；②*MAGE*基因表达与肺癌的病理分期有关，分期越晚，表达率越高；③*MAGE*基因表达与肺癌病理类型有关，在鳞癌中表达率高于肺癌。在检测时，各研究结果存在差异性，究其原因主要与检测技术、操作水平、指标选择等有关，鉴于*MAGE*基因在肺癌中的表达规律，*MAGE*基因表达检测可用于早期肺癌的筛选、诊断、分期评估及临床治疗。

影响肺癌预后的主要原因是血行转移和淋巴结转移，早期发现肺癌微转移将为早期诊断、指导临床治疗、检测疗效、判断肿瘤预后提供参考依据，但目前一般使用的检测标记物敏感性较低，迫切需要灵敏度高、特异性强的肿瘤标记物用于肺癌微转移检测。*MAGE*基因作为一种敏感度较高、特异性较强的标记物，越来越受到重视。刘涛等^[11]^采用RT-PCR技术，通过检测非小细胞肺癌患者外周血中的MAGE-1表达，研究非小细胞肺癌外周血微转移与临床特征之间的关系，结果显示*MAGE-1*基因在非小细胞肺癌患者外周血中阳性表达率为57.15%，远高于对照组中其阳性表达率（0/31）；外周血MAGE-1的表达与肿瘤分化程度、TNM分期、淋巴结转移关系密切。宋鉴清等^[12]^用甲基化特异性聚合酶链反应检测49例非小细胞肺癌患者外周血*MAGE-A1*、*MAGE-A3*基因启动子区CpG岛去甲基化的状态，分析与临床参数的关系，并进行24个月随访，结果肺癌患者外周血MAGE-A1和MAGE-A3启动子区CpG岛启动子呈去甲基化状态分别为53.1%和49%；对照组外周血标本均呈现甲基化（100%），通过随访发现肺癌组MAGE-A1、MAGE-A3启动子区CpG岛启动子呈去甲基化模式患者较呈甲基化患者预后不良，这说明MAGE-A1、MAGE-A3启动子区CpG岛启动子去甲基化模式与肺癌的转移、TNM分期及预后相关，通过对外周血相关基因的检测，可为肿瘤早期诊断、疗效观察及预后判断提供有价值的信息。在非小细胞肺癌淋巴结微转移检测方面，Dango等^[13]^将取自32例非小细胞肺癌患者术后的100枚淋巴结每枚平均分为两部分，分别进行常规病理检查及*MAGE-A*基因表达检测，常规病理检查阳性的淋巴结其*MAGE-A*基因表达率为66.7%，常规病理检查结果阴性的淋巴结*MAGE-A*基因表达阳性率为9.6%，共37.5%的肺癌患者淋巴结检测出*MAGE-A*基因表达，*MAGE-A*基因在淋巴结中的表达与肿瘤病理类型、病理分级、性别、年龄无关。Cucuruz等^[14]^运用LightCycler480仪器对89例肺癌患者的202枚淋巴结进行实时定量表达分析，结果提示MAGE-A1-6在支气管内超声引导下细针穿刺（endobronchial ultrasound-guided fine-needle aspiration, EBUS-TBNA）取得的样本中其表达率为28.7%，而在纵隔镜取得的样本中其表达率为20.0%；MAGE-A12在两种方式取得的样本中的表达率分别为8.2%和11.3%，经术后病理验证，术前检测到*MAGE*基因表达的患者，术后均诊断为广泛的区域淋巴结转移，术前EBUS-TBNA和纵隔镜结合MAGE定量聚合酶链反应，可将病理组织学诊断的准确性提高至81.2%和86.4%。冼磊等^[15]^采用RT-PCR方法检测53例非小细胞肺癌患者的111组淋巴结中MAGE-1、MAGE-2、MAGE-3、MAGE-4 mRNA的表达，其阳性表达率为41.4%，远高于常规病理检查结果的27.9%，常规病理检查结果阴性淋巴结中至少有1种*MAGE*基因表达的概率为23.8%，在常规病理检查结果阳性的淋巴结中其概率为87.1%，而在肺部良性疾病患者淋巴结中4种*MAGE*基因的表达均为0。相比于其它检测指标，*MAGE*基因具有高度特异性及敏感性，能更早地检测到非小细胞肺癌患者淋巴结、外周血中存在的微转移，不仅能更早地诊断肿瘤，还能更准确地估计肿瘤临床分期，为制定准确的治疗计划提供分子领域的支持。

### 2.3 *MAGE*基因在其它恶性肿瘤中的表达

*MAGE*基因不仅在肺癌中表达，在黑色素瘤、乳腺癌、肝癌、膀胱癌、大肠癌、胃癌、精原细胞瘤、头颈部鳞癌、食管癌、白血病、淋巴瘤和肉瘤等恶性肿瘤中均可检测到*MAGE*基因的表达。

Curioni-Fontecedro等^[16]^发现MAGE-C1在原发性黑色素瘤和和色素瘤细胞株中的阳性表达率同为24%，MAGE-C2在原发性黑色素瘤和和色素瘤细胞株中的阳性表达率分别为33%和14%，在远处转移瘤中MAGE-C1及MAGE-C2的阳性表达率均为40%，同时发现MAGE-C1或MAGE-C2阳性的原发黑色素瘤患者淋巴结转移率较高。Badovinac Črnjević等^[17]^发现乳腺癌患者肿瘤切片中MAGE-A10的阳性表达率为64%，其表达与肿瘤的大小、分级及淋巴结状态无关。Zhang等^[18]^运用实时-定量-聚合酶链反应发现肝癌患者外周血标本中MAGR-A1的阳性率为34.9%，AMGE-A3的阳性表达率为60.5%；在非肝癌患者血标本中并未检测到*MAGE*基因的阳性表达。Dyrskjøt等^[19]^对350例长期随访膀胱肿瘤患者的肿瘤标本行*MAGE*基因检测，结果显示43%的肿瘤表达*MAGE-A3*基因，56%的肿瘤至少表达1种CT基因，同时*MAGE-A3*基因的表达有预后的预测价值。

## 3 *MAGE*基因影响肿瘤生长的分子机制

虽然*MAGE*基因已经发现20余年，其生物学特性仍未清楚，但一些证据表明*MAGE*基因的表达与肿瘤的发生、发展及预后有密切的关系，可用于肿瘤的诊断和免疫治疗。首先，*MAGE*基因与肿瘤的发生有密切关系，主要是通过影响人体抑癌基因（*p53*基因）的活性来影响肿瘤发生。Yang等^[20]^研究发现MAGE-A、MAGE-B和MAGE-C等蛋白可能通过与*p53*基因结合，抑制其活性，促进肿瘤细胞的活化，促进恶性肿瘤的发生和发展。另有研究^[21]^显示MAGE-A可以与p53核心区的DNA结构域结合，阻断p53与启动子的结合，达到延长细胞周期，降低细胞死亡的作用，这项研究证明MAGE-A蛋白可以通过抑制p53的转录而引起肿瘤的发生发展。国内也可见类似报道，桑梅香等^[22]^发现转染*p53*基因可以使p21 WAF1启动子介导的荧光素酶表达增加，而共同转染恒定量的*p53*基因和不同量的*MAGE-A4*基因后p21 WAF1启动子介导的荧光素酶表达明显高于单独转染*p53*基因时的表达量；共同转染*MAGE-A4*基因和*p53*基因后p21 WAF1 mRNA和蛋白表达水平均明显高于单独转染*p53*基因时的表达水平；克隆形成实验及原位末端转移酶标记技术染色结果显示，共转染*MAGE-A4*基因和*p53*基因以后H1299细胞克隆形成数与单独转染*p53*基因组相比减少，而凋亡细胞数与单独转染*p53*基因组比较增加；最终结论证明MAGE-A4可以通过增强抑癌基因*p53*的转录活性，从而抑制肿瘤细胞的增殖并诱导肿瘤细胞凋亡。此外，MAGE-A3被发现是成纤维细胞生长因子受体2（fibroblast growth factor receptor 2, FGRF2）信号通路的靶基因，可负调节FGFR2的表达，并通过调节纤维连接蛋白而控制肿瘤的侵袭和转移^[23]^。*MAGE*基因还可通过细胞免疫达到杀灭肿瘤细胞的作用，在MAGE-A蛋白分子内至少有5个组织相容性复合体（major histocompatibility complex, MHC）Ⅰ类分子限制性表位和6个MHC Ⅱ类分子限制性表位，经由相应的MHC Ⅰ类或Ⅱ类分子递呈给辅助性T细胞（helper T cells, CD4^+^）和CD8^+^细胞，使人体产生针对相应肿瘤的细胞免疫。Hamaï等^[24]^研究发现，MAGE-A3抗肺癌机制为MAGE-A3抗原特定性CD4^+^细胞在白细胞介素17的影响下转化为效应T淋巴细胞，分泌干扰素，攻击并杀伤肿瘤细胞，达到抑制肺癌生长的作用。Baba等^[25]^研究发现MAGE-A抗原治疗肺癌的主要机制为其抗原首先作用于MHC基因簇，后控制人类白细胞抗原（human leukocyte antigen, HLA）Ⅰ类分子，将信息呈递给CD4^+^或CD8^+^细胞，产生干扰素或转变为效应T细胞，达到消灭肿瘤的作用。MAGE-A4表达阳性患者中HLA Ⅰ类分子缺乏患者的5年生存率明显低于HLA Ⅰ类分子健全患者。Peikert等^[26]^研究发现MAGE-A4稳定表达的细胞其凋亡指数和半胱氨酸天冬氨酸蛋白酶-3的活性均比MAGE-A4表达阴性的细胞高，在用MAGE-A4反义RNA干扰MAGE-A4表达后，细胞半胱氨酸天冬氨酸蛋白酶-3的活性明显降低，结果提示*MAGE-A4*基因参与细胞凋亡信号转导通路，可促进肿瘤细胞的凋亡，可能在肿瘤的发生中起到积极的防御作用，承载着治疗肿瘤的新希望。

## 4 *MAGE*基因编码蛋白的抗肿瘤治疗

以*MAGE*基因为靶点的抗癌作用主要通过以下3种方法：①接种MAGE表位抗原疫苗；②阻断*MAGE*基因与其配体的结合；③破坏操纵*MAGE*基因表达的监管系统。前两种方法可极大限度的限制MAGE蛋白在正常组织的表达，同时对机体影响较小、副作用较少，大多研究都着重于前两种方法。*MAGE*基因编码的相关抗原在正常组织不表达（睾丸和胎盘除外），而睾丸和胎盘是免疫豁免器官，因此MAGE相关抗原是肿瘤特异性免疫治疗的理想靶分子。

### 4.1 *MAGE*基因在恶性肿瘤免疫治疗的应用

*MAGE*基因抗肿瘤治疗要追溯到1999年，Marchand等^[27]^将多肽MAGE-A1和MAGE-A3为黑色素瘤患者皮下注射，接受治疗的25例患者中，7例肿瘤缩小，3例完全消失，2例的无瘤期达2年以上。经过不断探索研究，Glynn等^[28]^发现肿瘤细胞中只要表达*MAGE*基因，便可抵抗肿瘤坏死因子介导的细胞毒性作用，从而达到抑制肿瘤细胞凋亡的作用。之后，Ueda等^[29]^将MAGE-A1、MAGE-A3抗原肽用于食管原发性黑色素瘤患者的治疗，发现患者外周血中IFN-*γ*明显增多，患者的生存期较其它治疗方式有所提高。

### 4.2 *MAGE*基因在肺癌免疫治疗的应用

*MAGE*基因家族在肺癌中的广泛表达为抗肿瘤作用提供了潜在的靶点，目前相应的抗原性疫苗已经问世。国外有1例病例报道^[30]^，1位肺腺癌患者首次手术后复发，二次手术后再次复发，后接受MAGE-A3相关抗原免疫治疗，随访观察18个月未见复发迹象。国外一项随机Ⅱ期临床研究^[31]^将182例术后MAGE-A3表达阳性的肺癌患者（122例Ib期和60例Ⅱ期）随机分为两组，分别接受MAGE-A3疫苗和安慰剂治疗，中位随访时间为28个月，共发现67例复发病例和45例死亡病例，MAGE-A3疫苗组患者的无病间隔期、无病生存期及总生存率均优于对照组。另外开展的一项Ⅲ期临床研究^[32]^，预计全球招募2, 270例术后患者（Ib期-Ⅲa期），其主要目的是观察免疫治疗对肺癌术后复发的预防作用，此外还要观察免疫疗法的疗效，该研究在我国也招募患者，本项研究是目前世界最大的肺癌辅助治疗临床研究项目。

## 5 问题与展望

*MAGE*基因是较为敏感的肿瘤特异性基因，在肺癌肿瘤组织中高表达，而在非瘤性组织中罕有表达，在血液及正常淋巴结中无表达，在肺癌患者的外周血及转移淋巴结中高表达，其免疫产品具有特异性高、副作用小的特点，有望成为肺癌免疫治疗的靶点。加之肺癌是恶性肿瘤中发病率和病死率较高的肿瘤，*MAGE*基因与肺癌的关系已成为目前研究的热点，可在非小细胞肺癌的早期诊断、微转移灶检测、复发监测、免疫治疗及评估预后等工作中发挥作用。随着对*MAGE*基因家族在肺癌领域研究的不断深入，未来在肺癌的早期诊断、病理分级、临床分期、免疫治疗、评估预后等方面，*MAGE*基因家族将发挥更大的作用。
